# Di-μ-nicotinato-κ^2^ *N*:*O*;κ^2^ *O*:*N*-bis­[aqua­bis­(4-bromo­benz­yl)(nicotinato-κ^2^ *O*,*O*′)tin(IV)]

**DOI:** 10.1107/S1600536810028643

**Published:** 2010-07-24

**Authors:** Chun Thy Keng, Kong Mun Lo, Seik Weng Ng

**Affiliations:** a Department of Chemistry, University of Malaya, 50603 Kuala Lumpur, Malaysia

## Abstract

Two nicotinate binding modes are observed in the dinuclear title compound, [Sn_2_(C_7_H_6_Br)_4_(C_6_H_4_NO_2_)_4_(H_2_O)_2_]: in the first, a terminal *O*,*O*′-chelating molecule binds to a water-coordinated diorganotin cation, while the second mode corresponds to an *O*:*N*-bridging molecule which binds to two cations. The two Sn atoms exist in *trans*-C_2_SnNO_4_ penta­gonal-bipyramidal geometries. Adjacent dinuclear units are linked by O—H⋯N hydrogen bonds, generating a linear chain, which propagates in the *b*-axis direction. O—H⋯O inter­actions are also observed.

## Related literature

For the crystal structure of [Sn(C_7_H_6_F)_2_(C_6_H_4_NO_2_)]_2_, see: Yin *et al.* (2005 ▶).
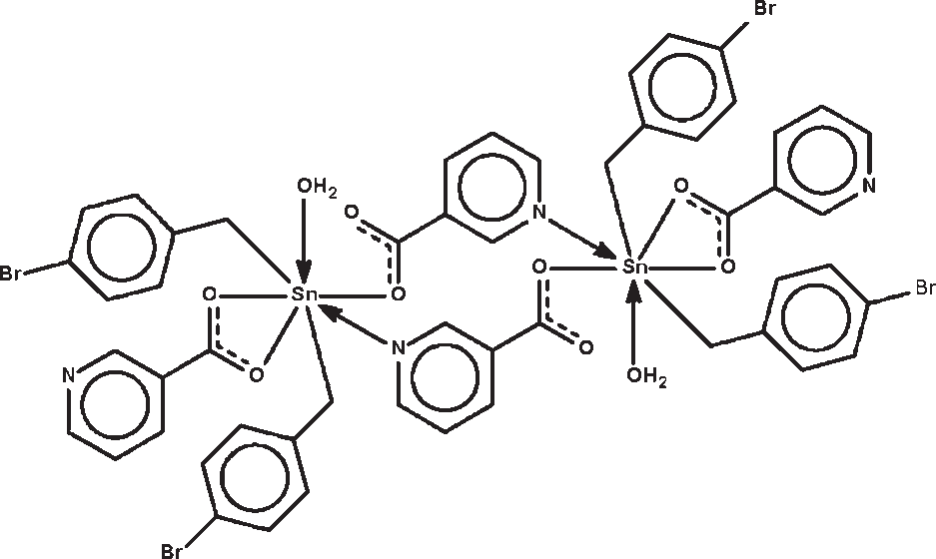


## Experimental

### 

#### Crystal data

[Sn_2_(C_7_H_6_Br)_4_(C_6_H_4_NO_2_)_4_(H_2_O)_2_]
*M* *_r_* = 1441.93
Monoclinic, 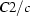

*a* = 28.3754 (13) Å
*b* = 16.7568 (7) Å
*c* = 21.5642 (10) Å
β = 90.998 (1)°
*V* = 10251.8 (8) Å^3^
*Z* = 8
Mo *K*α radiation
μ = 4.16 mm^−1^
*T* = 100 K
0.45 × 0.15 × 0.15 mm

#### Data collection

Bruker SMART APEX diffractometer
Absorption correction: multi-scan (*SADABS*; Sheldrick, 1996 ▶) *T*_min_ = 0.256, *T*_max_ = 0.575
48701 measured reflections
11790 independent reflections
9879 reflections with *I* > 2σ(*I*)
*R*_int_ = 0.040

#### Refinement

*R*[*F* ^2^ > 2σ(*F* ^2^)] = 0.028
*wR*(*F* ^2^) = 0.066
*S* = 1.18
11790 reflections
649 parameters
H-atom parameters constrained
Δρ_max_ = 1.71 e Å^−3^
Δρ_min_ = −1.35 e Å^−3^

### 

Data collection: *APEX2* (Bruker, 2009 ▶); cell refinement: *SAINT* (Bruker, 2009 ▶); data reduction: *SAINT*; program(s) used to solve structure: *SHELXS97* (Sheldrick, 2008 ▶); program(s) used to refine structure: *SHELXL97* (Sheldrick, 2008 ▶); molecular graphics: *X-SEED* (Barbour, 2001 ▶); software used to prepare material for publication: *publCIF* (Westrip, 2010 ▶).

## Supplementary Material

Crystal structure: contains datablocks global, I. DOI: 10.1107/S1600536810028643/nk2048sup1.cif

Structure factors: contains datablocks I. DOI: 10.1107/S1600536810028643/nk2048Isup2.hkl

Additional supplementary materials:  crystallographic information; 3D view; checkCIF report

## Figures and Tables

**Table 1:** Selected bond angles (°)

C1—Sn1—C8	175.5 (1)
C27—Sn2—C34	175.2 (1)

**Table 2:** Hydrogen-bond geometry (Å, °)

*D*—H⋯*A*	*D*—H	H⋯*A*	*D*⋯*A*	*D*—H⋯*A*
O1*w*—H1*w*1⋯O4	0.84	1.94	2.557 (2)	129
O1*w*—H1*w*2⋯N3^i^	0.84	2.06	2.663 (3)	128
O2*w*—H2*w*2⋯O8	0.84	1.96	2.563 (2)	128
O2*w*—H2*w*1⋯N1^ii^	0.84	2.26	2.690 (3)	112

Symmetry codes: (i) 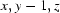; (ii) 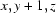.

**Fig. 1.**
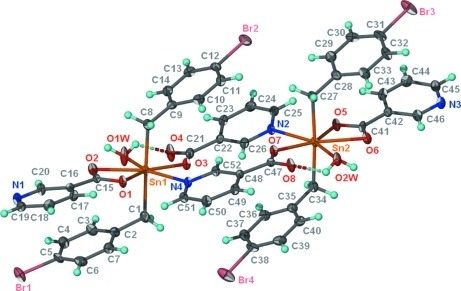
Displacement ellipsoid plot of [Sn(H2O)(C7H6Br)2(C6H4NO2)]2; ellipsoids are drawn at the 70% probability level and H atoms are of arbitrary radius.

## supplementary crystallographic information

### Comment

Diorganotin dicarboxylates generally have their tin atoms in a six-coordinate skew-trapezoidal bipyramidal geometry; however, for the carboxylate unit such as the nicotinate ion that possesses a Lewis-basic nitrogen donor-atom, the nitrogen atom can participate in intermolecular coordination. This is exemplified by bis(4-fluorobenzyl)di(nicotinato)tin in which both nicotinate ions *O*,*O*'-chelate to the tin atom. However, the coordination number is raised to seven owing to the interaction of one of the two pyridyl nitrogen atoms; this tin-nitrogen bond gives rise to the formation of a chain coordination polymer (Yin *et al.*, 2005). The title bromo analog (Scheme I) is not, however, isomorphous as it crystallizes as a water-coordinated, dinuclear compound (Fig. 1). One nicotinate ion functions in an *O*,*O*'-chelating mode whereas the other nicotinate ion functions in an *O*:*N*'-bridging mode. The two seven-coordinate tin atoms show *trans*-C_2_SnNO_4_ pentagonal bipyramidal geometry; the C_2_Sn skeletons are nearly linear (Table 1).

### Experimental

Di(4-bromobenzyl)tin oxide (0.40 g, 0.84 mmol) was suspended in chloroform (20 ml) and to the suspension was added an ethanol (20 ml) solution of nicotinic acid (0.21 g, 1.68 mmol). The mixture was heated for three hours; the solution was filtere and the solvent allow to evaporate. Colorless crystals were isolated after several days.

### Refinement

Hydrogen atoms were placed in calculated positions (C–H 0.95–0.99, O–H 0.84 Å) and were included in the refinement in the riding model approximation, with *U*(H) set to 1.2–1.5*U*_eq_(C,*O*). The final difference Fourier map had a peak of 1.7 *e* Å^-3^ at 0.83 Å from Br3 and a hole of -1.4 *e* Å^-3^ at 0.7 Å from Br3.

### Crystal data

**Table:**

[Sn_2_(C_7_H_6_Br)_4_(C_6_H_4_NO_2_)_4_(H_2_O)_2_]	*F*(000) = 5632
*M**_r_* = 1441.93	*D*_x_ = 1.868 Mg m^−^^3^
Monoclinic, *C*2/*c*	Mo *K*α radiation, λ = 0.71073 Å
Hall symbol: -C 2yc	Cell parameters from 9852 reflections
*a* = 28.3754 (13) Å	θ = 2.4–28.3°
*b* = 16.7568 (7) Å	µ = 4.16 mm^−^^1^
*c* = 21.5642 (10) Å	*T* = 100 K
β = 90.998 (1)°	Block, colourless
*V* = 10251.8 (8) Å^3^	0.45 × 0.15 × 0.15 mm
*Z* = 8	

### Data collection

**Table:**

Bruker SMART APEX diffractometer	11790 independent reflections
Radiation source: fine-focus sealed tube	9879 reflections with *I* > 2σ(*I*)
graphite	*R*_int_ = 0.040
ω scans	θ_max_ = 27.5°, θ_min_ = 1.4°
Absorption correction: multi-scan (*SADABS*; Sheldrick, 1996)	*h* = −36→36
*T*_min_ = 0.256, *T*_max_ = 0.575	*k* = −21→21
48701 measured reflections	*l* = −28→28

### Refinement

**Table:**

Refinement on *F*^2^	Primary atom site location: structure-invariant direct methods
Least-squares matrix: full	Secondary atom site location: difference Fourier map
*R*[*F*^2^ > 2σ(*F*^2^)] = 0.028	Hydrogen site location: inferred from neighbouring sites
*wR*(*F*^2^) = 0.066	H-atom parameters constrained
*S* = 1.18	*w* = 1/[σ^2^(*F*_o_^2^) + (0.0282*P*)^2^] where *P* = (*F*_o_^2^ + 2*F*_c_^2^)/3
11790 reflections	(Δ/σ)_max_ = 0.001
649 parameters	Δρ_max_ = 1.71 e Å^−^^3^
0 restraints	Δρ_min_ = −1.35 e Å^−^^3^

### Fractional atomic coordinates and isotropic or equivalent isotropic displacement parameters (Å^2^)

**Table:**

	*x*	*y*	*z*	*U*_iso_*/*U*_eq_	
Sn1	0.127365 (6)	0.398907 (10)	0.475980 (8)	0.01245 (5)	
Sn2	0.118973 (6)	0.852588 (10)	0.497094 (8)	0.01254 (5)	
Br1	0.061186 (11)	0.031350 (17)	0.317354 (15)	0.02867 (8)	
Br2	0.184067 (10)	0.707467 (17)	0.714509 (13)	0.02448 (7)	
Br3	0.189153 (11)	1.149300 (19)	0.736615 (16)	0.03382 (8)	
Br4	0.068462 (11)	0.57137 (2)	0.243172 (15)	0.03221 (8)	
O1	0.18011 (6)	0.33219 (10)	0.41027 (8)	0.0168 (4)	
O2	0.14491 (6)	0.26003 (10)	0.48040 (8)	0.0171 (4)	
O3	0.09053 (6)	0.50964 (10)	0.50952 (8)	0.0151 (4)	
O4	0.04097 (7)	0.47396 (10)	0.58468 (10)	0.0247 (5)	
O5	0.07314 (6)	0.91872 (10)	0.57150 (9)	0.0178 (4)	
O6	0.10635 (6)	0.99148 (10)	0.50002 (8)	0.0174 (4)	
O7	0.15283 (6)	0.74040 (10)	0.45926 (8)	0.0148 (4)	
O8	0.19850 (6)	0.77716 (10)	0.37971 (9)	0.0187 (4)	
O1w	0.08643 (7)	0.34945 (11)	0.55222 (10)	0.0286 (5)	
H1w1	0.0838	0.3840	0.5802	0.043*	
H1w2	0.1003	0.3091	0.5667	0.043*	
O2w	0.15534 (6)	0.90247 (10)	0.41642 (8)	0.0193 (4)	
H2w1	0.1409	0.9432	0.4037	0.029*	
H2w2	0.1559	0.8683	0.3880	0.029*	
N1	0.18947 (8)	0.04941 (12)	0.39462 (10)	0.0170 (5)	
N2	0.08851 (7)	0.74508 (12)	0.56837 (10)	0.0146 (4)	
N3	0.07093 (8)	1.20199 (12)	0.59318 (10)	0.0177 (5)	
N4	0.15900 (7)	0.50348 (12)	0.40569 (10)	0.0140 (4)	
C1	0.07051 (9)	0.38070 (15)	0.41051 (13)	0.0203 (6)	
H1A	0.0406	0.3957	0.4304	0.024*	
H1B	0.0749	0.4169	0.3748	0.024*	
C2	0.06617 (9)	0.29671 (15)	0.38672 (13)	0.0171 (5)	
C3	0.04277 (9)	0.23804 (16)	0.41982 (13)	0.0197 (6)	
H3	0.0279	0.2522	0.4574	0.024*	
C4	0.04046 (9)	0.15967 (16)	0.39964 (13)	0.0192 (6)	
H4	0.0243	0.1205	0.4230	0.023*	
C5	0.06211 (9)	0.13949 (15)	0.34497 (13)	0.0190 (6)	
C6	0.08511 (10)	0.19585 (16)	0.31007 (12)	0.0210 (6)	
H6	0.0994	0.1814	0.2721	0.025*	
C7	0.08709 (10)	0.27414 (16)	0.33135 (13)	0.0202 (6)	
H7	0.1031	0.3131	0.3076	0.024*	
C8	0.18753 (9)	0.41961 (15)	0.53542 (12)	0.0169 (5)	
H8A	0.1926	0.3713	0.5611	0.020*	
H8B	0.2155	0.4258	0.5090	0.020*	
C9	0.18555 (9)	0.49006 (15)	0.57785 (12)	0.0150 (5)	
C10	0.20789 (9)	0.56172 (15)	0.56315 (12)	0.0179 (6)	
H10	0.2239	0.5661	0.5250	0.021*	
C11	0.20727 (9)	0.62685 (15)	0.60305 (13)	0.0174 (6)	
H11	0.2226	0.6752	0.5924	0.021*	
C12	0.18395 (9)	0.61987 (15)	0.65828 (12)	0.0168 (5)	
C13	0.16116 (9)	0.55024 (16)	0.67453 (12)	0.0181 (6)	
H13	0.1452	0.5465	0.7128	0.022*	
C14	0.16198 (9)	0.48563 (15)	0.63386 (12)	0.0167 (5)	
H14	0.1462	0.4377	0.6445	0.020*	
C15	0.17112 (8)	0.26564 (14)	0.43434 (12)	0.0123 (5)	
C16	0.19032 (9)	0.19214 (14)	0.40448 (11)	0.0126 (5)	
C17	0.22184 (9)	0.19773 (15)	0.35631 (12)	0.0165 (5)	
H17	0.2329	0.2483	0.3431	0.020*	
C18	0.23693 (10)	0.12886 (15)	0.32781 (12)	0.0187 (6)	
H18	0.2587	0.1312	0.2949	0.022*	
C19	0.21978 (9)	0.05604 (15)	0.34813 (12)	0.0176 (6)	
H19	0.2300	0.0088	0.3281	0.021*	
C20	0.17523 (9)	0.11638 (15)	0.42222 (13)	0.0162 (5)	
H20	0.1538	0.1122	0.4555	0.019*	
C21	0.06516 (9)	0.52344 (15)	0.55653 (12)	0.0148 (5)	
C22	0.06603 (8)	0.60797 (14)	0.58108 (12)	0.0131 (5)	
C23	0.04915 (9)	0.62469 (15)	0.64008 (12)	0.0156 (5)	
H23	0.0353	0.5840	0.6644	0.019*	
C24	0.05306 (9)	0.70185 (15)	0.66245 (12)	0.0182 (6)	
H24	0.0422	0.7146	0.7027	0.022*	
C25	0.07298 (9)	0.76045 (15)	0.62577 (12)	0.0163 (5)	
H25	0.0757	0.8131	0.6417	0.020*	
C26	0.08499 (9)	0.66972 (14)	0.54711 (12)	0.0148 (5)	
H26	0.0961	0.6584	0.5067	0.018*	
C27	0.18036 (9)	0.86188 (15)	0.55646 (13)	0.0174 (6)	
H27A	0.1824	0.8128	0.5820	0.021*	
H27B	0.2085	0.8630	0.5299	0.021*	
C28	0.18292 (9)	0.93245 (15)	0.59928 (12)	0.0166 (5)	
C29	0.16707 (9)	0.92619 (16)	0.66014 (13)	0.0197 (6)	
H29	0.1553	0.8765	0.6743	0.024*	
C30	0.16818 (9)	0.99067 (17)	0.70021 (13)	0.0212 (6)	
H30	0.1568	0.9857	0.7412	0.025*	
C31	0.18609 (9)	1.06259 (16)	0.67960 (13)	0.0210 (6)	
C32	0.20203 (10)	1.07127 (16)	0.61970 (14)	0.0226 (6)	
H32	0.2140	1.1210	0.6059	0.027*	
C33	0.20016 (9)	1.00592 (15)	0.58009 (13)	0.0190 (6)	
H33	0.2109	1.0116	0.5388	0.023*	
C34	0.05569 (9)	0.83499 (15)	0.44248 (13)	0.0179 (6)	
H34A	0.0297	0.8220	0.4708	0.021*	
H34B	0.0476	0.8859	0.4216	0.021*	
C35	0.05825 (9)	0.77112 (15)	0.39438 (12)	0.0159 (5)	
C36	0.03717 (9)	0.69641 (15)	0.40305 (12)	0.0174 (5)	
H36	0.0205	0.6866	0.4401	0.021*	
C37	0.04008 (9)	0.63636 (15)	0.35879 (13)	0.0175 (6)	
H37	0.0254	0.5862	0.3651	0.021*	
C38	0.06478 (9)	0.65132 (16)	0.30563 (13)	0.0192 (6)	
C39	0.08604 (9)	0.72418 (16)	0.29506 (12)	0.0203 (6)	
H39	0.1029	0.7333	0.2581	0.024*	
C40	0.08231 (9)	0.78390 (16)	0.33935 (12)	0.0187 (6)	
H40	0.0964	0.8344	0.3321	0.022*	
C41	0.08379 (9)	0.98551 (14)	0.54961 (12)	0.0132 (5)	
C42	0.07266 (9)	1.05913 (14)	0.58653 (12)	0.0135 (5)	
C43	0.05598 (9)	1.05319 (15)	0.64635 (12)	0.0178 (6)	
H43	0.0508	1.0024	0.6646	0.021*	
C44	0.04701 (10)	1.12212 (16)	0.67904 (13)	0.0202 (6)	
H44	0.0359	1.1197	0.7203	0.024*	
C45	0.05458 (9)	1.19507 (15)	0.65050 (12)	0.0182 (6)	
H45	0.0478	1.2424	0.6729	0.022*	
C46	0.08032 (9)	1.13463 (14)	0.56181 (12)	0.0162 (5)	
H46	0.0926	1.1388	0.5213	0.019*	
C47	0.17733 (8)	0.72694 (14)	0.41137 (11)	0.0125 (5)	
C48	0.17991 (8)	0.64110 (14)	0.39044 (12)	0.0120 (5)	
C49	0.20248 (9)	0.62168 (15)	0.33556 (12)	0.0165 (5)	
H49	0.2177	0.6616	0.3119	0.020*	
C50	0.20213 (9)	0.54268 (15)	0.31627 (12)	0.0181 (6)	
H50	0.2171	0.5278	0.2790	0.022*	
C51	0.17979 (9)	0.48584 (15)	0.35180 (12)	0.0161 (5)	
H51	0.1791	0.4322	0.3376	0.019*	
C52	0.15920 (9)	0.58055 (14)	0.42374 (12)	0.0136 (5)	
H52	0.1443	0.5939	0.4614	0.016*	

### Atomic displacement parameters (Å^2^)

**Table:**

	*U*^11^	*U*^22^	*U*^33^	*U*^12^	*U*^13^	*U*^23^
Sn1	0.01387 (9)	0.00750 (8)	0.01606 (9)	−0.00060 (6)	0.00270 (7)	−0.00051 (6)
Sn2	0.01477 (9)	0.00796 (8)	0.01494 (9)	−0.00121 (6)	0.00193 (7)	−0.00129 (6)
Br1	0.03283 (17)	0.01812 (14)	0.03515 (18)	−0.00559 (12)	0.00369 (14)	−0.00993 (12)
Br2	0.02351 (15)	0.02344 (15)	0.02635 (16)	0.00356 (11)	−0.00397 (12)	−0.01179 (12)
Br3	0.02835 (17)	0.03383 (17)	0.03937 (19)	−0.00471 (13)	0.00374 (14)	−0.02383 (14)
Br4	0.02297 (16)	0.04017 (18)	0.03365 (18)	−0.00338 (13)	0.00543 (13)	−0.02233 (14)
O1	0.0216 (10)	0.0087 (8)	0.0202 (10)	−0.0004 (7)	0.0031 (8)	0.0001 (7)
O2	0.0173 (9)	0.0133 (9)	0.0207 (10)	0.0005 (7)	0.0049 (8)	−0.0019 (7)
O3	0.0169 (9)	0.0104 (8)	0.0180 (10)	0.0006 (7)	0.0054 (7)	0.0010 (7)
O4	0.0260 (11)	0.0105 (9)	0.0381 (12)	−0.0027 (8)	0.0181 (9)	0.0006 (8)
O5	0.0205 (10)	0.0099 (9)	0.0230 (10)	−0.0013 (7)	0.0034 (8)	−0.0003 (7)
O6	0.0212 (10)	0.0126 (9)	0.0186 (10)	−0.0014 (7)	0.0032 (8)	−0.0014 (7)
O7	0.0186 (9)	0.0098 (8)	0.0163 (9)	−0.0012 (7)	0.0034 (7)	−0.0003 (7)
O8	0.0217 (10)	0.0099 (9)	0.0249 (10)	−0.0037 (7)	0.0083 (8)	−0.0006 (7)
O1w	0.0395 (13)	0.0098 (9)	0.0374 (13)	0.0066 (8)	0.0227 (10)	0.0025 (8)
O2w	0.0272 (11)	0.0101 (9)	0.0208 (10)	0.0014 (8)	0.0073 (8)	−0.0015 (7)
N1	0.0183 (12)	0.0110 (10)	0.0218 (12)	0.0016 (9)	0.0020 (9)	−0.0003 (9)
N2	0.0155 (11)	0.0094 (10)	0.0190 (12)	−0.0004 (8)	0.0004 (9)	−0.0005 (8)
N3	0.0205 (12)	0.0118 (11)	0.0210 (12)	0.0010 (9)	0.0026 (9)	0.0000 (9)
N4	0.0163 (11)	0.0097 (10)	0.0161 (11)	0.0002 (8)	0.0013 (9)	0.0007 (8)
C1	0.0174 (14)	0.0140 (13)	0.0294 (16)	−0.0026 (10)	−0.0043 (12)	−0.0010 (11)
C2	0.0129 (13)	0.0153 (13)	0.0228 (14)	−0.0016 (10)	−0.0045 (11)	−0.0009 (11)
C3	0.0186 (14)	0.0198 (14)	0.0210 (14)	−0.0030 (11)	0.0033 (11)	−0.0037 (11)
C4	0.0173 (14)	0.0173 (13)	0.0231 (15)	−0.0058 (11)	0.0015 (11)	0.0007 (11)
C5	0.0203 (14)	0.0142 (13)	0.0224 (15)	−0.0040 (11)	−0.0052 (12)	−0.0063 (11)
C6	0.0240 (15)	0.0246 (15)	0.0143 (13)	−0.0030 (12)	0.0000 (11)	−0.0041 (11)
C7	0.0227 (15)	0.0180 (13)	0.0198 (14)	−0.0063 (11)	−0.0011 (11)	0.0041 (11)
C8	0.0174 (13)	0.0138 (12)	0.0194 (14)	0.0012 (10)	0.0008 (11)	−0.0022 (10)
C9	0.0124 (13)	0.0160 (13)	0.0164 (13)	0.0018 (10)	−0.0032 (10)	−0.0007 (10)
C10	0.0161 (13)	0.0214 (14)	0.0162 (13)	−0.0015 (11)	0.0005 (11)	0.0004 (11)
C11	0.0135 (13)	0.0146 (13)	0.0241 (15)	−0.0001 (10)	−0.0007 (11)	0.0019 (11)
C12	0.0144 (13)	0.0177 (13)	0.0180 (14)	0.0044 (10)	−0.0049 (11)	−0.0063 (10)
C13	0.0162 (13)	0.0227 (14)	0.0153 (13)	0.0033 (11)	0.0010 (11)	0.0005 (11)
C14	0.0140 (13)	0.0163 (13)	0.0198 (14)	0.0006 (10)	−0.0008 (11)	0.0011 (11)
C15	0.0096 (12)	0.0091 (11)	0.0182 (13)	0.0004 (9)	−0.0026 (10)	−0.0010 (10)
C16	0.0140 (12)	0.0098 (12)	0.0138 (12)	0.0013 (9)	−0.0011 (10)	−0.0007 (10)
C17	0.0188 (14)	0.0123 (12)	0.0182 (13)	−0.0011 (10)	0.0000 (11)	0.0028 (10)
C18	0.0221 (14)	0.0196 (14)	0.0144 (13)	0.0018 (11)	0.0050 (11)	0.0017 (10)
C19	0.0225 (14)	0.0138 (13)	0.0165 (13)	0.0043 (11)	−0.0029 (11)	−0.0023 (10)
C20	0.0159 (13)	0.0119 (12)	0.0210 (14)	−0.0001 (10)	0.0036 (11)	0.0000 (10)
C21	0.0130 (12)	0.0115 (12)	0.0198 (14)	0.0019 (10)	0.0002 (10)	0.0004 (10)
C22	0.0112 (12)	0.0106 (12)	0.0174 (13)	0.0030 (9)	−0.0015 (10)	0.0008 (10)
C23	0.0155 (13)	0.0141 (12)	0.0170 (13)	0.0015 (10)	0.0022 (11)	0.0038 (10)
C24	0.0209 (14)	0.0205 (14)	0.0131 (13)	0.0014 (11)	0.0014 (11)	0.0014 (10)
C25	0.0203 (14)	0.0127 (12)	0.0160 (13)	0.0009 (10)	−0.0008 (11)	−0.0007 (10)
C26	0.0136 (12)	0.0133 (12)	0.0177 (13)	0.0018 (10)	0.0022 (10)	0.0002 (10)
C27	0.0174 (13)	0.0131 (12)	0.0218 (14)	−0.0009 (10)	0.0002 (11)	−0.0033 (10)
C28	0.0133 (13)	0.0163 (13)	0.0201 (14)	0.0001 (10)	−0.0020 (11)	−0.0031 (11)
C29	0.0188 (14)	0.0190 (13)	0.0214 (14)	−0.0017 (11)	−0.0008 (11)	0.0019 (11)
C30	0.0187 (14)	0.0290 (15)	0.0160 (14)	−0.0010 (12)	−0.0017 (11)	−0.0036 (11)
C31	0.0167 (14)	0.0225 (14)	0.0238 (15)	0.0009 (11)	−0.0024 (11)	−0.0114 (12)
C32	0.0213 (15)	0.0162 (13)	0.0301 (16)	−0.0044 (11)	0.0004 (12)	−0.0049 (12)
C33	0.0196 (14)	0.0188 (13)	0.0188 (14)	−0.0026 (11)	0.0031 (11)	−0.0042 (11)
C34	0.0175 (14)	0.0140 (13)	0.0222 (14)	0.0000 (10)	0.0004 (11)	−0.0009 (11)
C35	0.0122 (12)	0.0168 (13)	0.0186 (13)	−0.0004 (10)	−0.0020 (10)	−0.0018 (10)
C36	0.0150 (13)	0.0200 (13)	0.0171 (13)	−0.0016 (10)	0.0016 (10)	0.0005 (11)
C37	0.0134 (13)	0.0163 (13)	0.0229 (15)	−0.0020 (10)	0.0008 (11)	−0.0013 (11)
C38	0.0163 (13)	0.0225 (14)	0.0187 (14)	0.0017 (11)	−0.0031 (11)	−0.0081 (11)
C39	0.0174 (14)	0.0301 (15)	0.0132 (13)	−0.0045 (12)	0.0005 (11)	0.0018 (11)
C40	0.0188 (14)	0.0187 (13)	0.0185 (14)	−0.0029 (11)	−0.0026 (11)	0.0036 (11)
C41	0.0119 (12)	0.0087 (11)	0.0190 (13)	0.0029 (9)	0.0019 (10)	0.0026 (10)
C42	0.0125 (12)	0.0115 (12)	0.0166 (13)	0.0009 (10)	−0.0005 (10)	0.0003 (10)
C43	0.0199 (14)	0.0139 (12)	0.0198 (14)	0.0014 (10)	0.0034 (11)	0.0039 (10)
C44	0.0229 (15)	0.0214 (14)	0.0163 (14)	0.0023 (11)	0.0044 (11)	0.0002 (11)
C45	0.0205 (14)	0.0142 (13)	0.0200 (14)	0.0038 (10)	0.0009 (11)	−0.0044 (11)
C46	0.0179 (13)	0.0140 (13)	0.0169 (14)	0.0000 (10)	0.0041 (11)	−0.0007 (10)
C47	0.0119 (12)	0.0105 (12)	0.0151 (13)	−0.0008 (9)	−0.0021 (10)	0.0001 (10)
C48	0.0115 (12)	0.0098 (12)	0.0148 (13)	0.0009 (9)	−0.0012 (10)	−0.0001 (9)
C49	0.0172 (13)	0.0133 (12)	0.0190 (14)	−0.0013 (10)	0.0010 (11)	0.0023 (10)
C50	0.0247 (15)	0.0155 (13)	0.0143 (13)	0.0010 (11)	0.0050 (11)	−0.0008 (10)
C51	0.0205 (14)	0.0120 (12)	0.0158 (13)	0.0016 (10)	0.0003 (11)	−0.0026 (10)
C52	0.0127 (12)	0.0111 (12)	0.0171 (13)	0.0005 (9)	0.0017 (10)	−0.0006 (10)

### Geometric parameters (Å, °)

**Table:**

Sn1—C8	2.145 (3)	C14—H14	0.9500
Sn1—C1	2.147 (3)	C15—C16	1.497 (3)
Sn1—O1w	2.1922 (18)	C16—C17	1.386 (3)
Sn1—O3	2.2549 (17)	C16—C20	1.395 (3)
Sn1—O1	2.3605 (17)	C17—C18	1.379 (4)
Sn1—O2	2.3813 (17)	C17—H17	0.9500
Sn1—N4	2.494 (2)	C18—C19	1.387 (4)
Sn2—C27	2.149 (3)	C18—H18	0.9500
Sn2—C34	2.150 (3)	C19—H19	0.9500
Sn2—O2w	2.2029 (17)	C20—H20	0.9500
Sn2—O7	2.2695 (16)	C21—C22	1.512 (3)
Sn2—O6	2.3558 (17)	C22—C26	1.382 (3)
Sn2—O5	2.3594 (17)	C22—C23	1.396 (3)
Sn2—N2	2.530 (2)	C23—C24	1.384 (4)
Br1—C5	1.907 (3)	C23—H23	0.9500
Br2—C12	1.904 (3)	C24—C25	1.387 (3)
Br3—C31	1.904 (3)	C24—H24	0.9500
Br4—C38	1.904 (3)	C25—H25	0.9500
O1—C15	1.258 (3)	C26—H26	0.9500
O2—C15	1.255 (3)	C27—C28	1.501 (3)
O3—C21	1.275 (3)	C27—H27A	0.9900
O4—C21	1.242 (3)	C27—H27B	0.9900
O5—C41	1.254 (3)	C28—C33	1.390 (4)
O6—C41	1.260 (3)	C28—C29	1.399 (4)
O7—C47	1.275 (3)	C29—C30	1.383 (4)
O8—C47	1.245 (3)	C29—H29	0.9500
O1w—H1w1	0.8400	C30—C31	1.384 (4)
O1w—H1w2	0.8400	C30—H30	0.9500
O2w—H2w1	0.8400	C31—C32	1.384 (4)
O2w—H2w2	0.8400	C32—C33	1.389 (4)
N1—C20	1.336 (3)	C32—H32	0.9500
N1—C19	1.337 (3)	C33—H33	0.9500
N2—C26	1.347 (3)	C34—C35	1.493 (3)
N2—C25	1.346 (3)	C34—H34A	0.9900
N3—C45	1.333 (3)	C34—H34B	0.9900
N3—C46	1.345 (3)	C35—C40	1.396 (4)
N4—C51	1.345 (3)	C35—C36	1.401 (4)
N4—C52	1.349 (3)	C36—C37	1.390 (4)
C1—C2	1.502 (4)	C36—H36	0.9500
C1—H1A	0.9900	C37—C38	1.377 (4)
C1—H1B	0.9900	C37—H37	0.9500
C2—C3	1.390 (4)	C38—C39	1.382 (4)
C2—C7	1.395 (4)	C39—C40	1.389 (4)
C3—C4	1.385 (4)	C39—H39	0.9500
C3—H3	0.9500	C40—H40	0.9500
C4—C5	1.381 (4)	C41—C42	1.505 (3)
C4—H4	0.9500	C42—C43	1.385 (3)
C5—C6	1.379 (4)	C42—C46	1.391 (3)
C6—C7	1.391 (4)	C43—C44	1.379 (4)
C6—H6	0.9500	C43—H43	0.9500
C7—H7	0.9500	C44—C45	1.387 (4)
C8—C9	1.495 (3)	C44—H44	0.9500
C8—H8A	0.9900	C45—H45	0.9500
C8—H8B	0.9900	C46—H46	0.9500
C9—C14	1.393 (3)	C47—C48	1.510 (3)
C9—C10	1.397 (4)	C48—C52	1.380 (3)
C10—C11	1.390 (4)	C48—C49	1.394 (3)
C10—H10	0.9500	C49—C50	1.387 (3)
C11—C12	1.377 (4)	C49—H49	0.9500
C11—H11	0.9500	C50—C51	1.383 (3)
C12—C13	1.382 (4)	C50—H50	0.9500
C13—C14	1.394 (4)	C51—H51	0.9500
C13—H13	0.9500	C52—H52	0.9500
			
C1—Sn1—C8	175.5 (1)	C20—C16—C15	121.0 (2)
C8—Sn1—O1w	92.27 (9)	C18—C17—C16	119.1 (2)
C1—Sn1—O1w	92.15 (10)	C18—C17—H17	120.4
C8—Sn1—O3	92.50 (8)	C16—C17—H17	120.4
C1—Sn1—O3	88.89 (8)	C17—C18—C19	118.8 (2)
O1w—Sn1—O3	79.39 (6)	C17—C18—H18	120.6
C8—Sn1—O1	85.93 (8)	C19—C18—H18	120.6
C1—Sn1—O1	90.86 (9)	N1—C19—C18	122.9 (2)
O1w—Sn1—O1	128.39 (6)	N1—C19—H19	118.5
O3—Sn1—O1	152.19 (6)	C18—C19—H19	118.5
C8—Sn1—O2	88.31 (8)	N1—C20—C16	123.1 (2)
C1—Sn1—O2	92.39 (8)	N1—C20—H20	118.5
O1w—Sn1—O2	73.36 (6)	C16—C20—H20	118.5
O3—Sn1—O2	152.75 (6)	O4—C21—O3	126.3 (2)
O1—Sn1—O2	55.04 (6)	O4—C21—C22	117.4 (2)
C8—Sn1—N4	87.61 (9)	O3—C21—C22	116.2 (2)
C1—Sn1—N4	88.54 (9)	C26—C22—C23	118.4 (2)
O1w—Sn1—N4	157.27 (7)	C26—C22—C21	121.3 (2)
O3—Sn1—N4	77.91 (6)	C23—C22—C21	120.2 (2)
O1—Sn1—N4	74.29 (6)	C24—C23—C22	118.6 (2)
O2—Sn1—N4	129.32 (6)	C24—C23—H23	120.7
C27—Sn2—C34	175.2 (1)	C22—C23—H23	120.7
C27—Sn2—O2w	93.27 (9)	C23—C24—C25	119.6 (2)
C34—Sn2—O2w	91.01 (9)	C23—C24—H24	120.2
C27—Sn2—O7	86.01 (8)	C25—C24—H24	120.2
C34—Sn2—O7	92.53 (8)	N2—C25—C24	122.1 (2)
O2w—Sn2—O7	79.82 (6)	N2—C25—H25	118.9
C27—Sn2—O6	91.98 (8)	C24—C25—H25	118.9
C34—Sn2—O6	91.39 (8)	N2—C26—C22	123.3 (2)
O2w—Sn2—O6	73.73 (6)	N2—C26—H26	118.4
O7—Sn2—O6	153.32 (6)	C22—C26—H26	118.4
C27—Sn2—O5	90.66 (8)	C28—C27—Sn2	116.96 (18)
C34—Sn2—O5	88.39 (8)	C28—C27—H27A	108.1
O2w—Sn2—O5	129.06 (6)	Sn2—C27—H27A	108.1
O7—Sn2—O5	151.09 (6)	C28—C27—H27B	108.1
O6—Sn2—O5	55.38 (6)	Sn2—C27—H27B	108.1
C27—Sn2—N2	88.38 (9)	H27A—C27—H27B	107.3
C34—Sn2—N2	86.80 (9)	C33—C28—C29	117.8 (2)
O2w—Sn2—N2	156.88 (6)	C33—C28—C27	121.9 (2)
O7—Sn2—N2	77.28 (6)	C29—C28—C27	120.3 (2)
O6—Sn2—N2	129.30 (6)	C30—C29—C28	121.6 (2)
O5—Sn2—N2	73.92 (6)	C30—C29—H29	119.2
C15—O1—Sn1	92.11 (14)	C28—C29—H29	119.2
C15—O2—Sn1	91.23 (14)	C29—C30—C31	118.9 (3)
C21—O3—Sn1	132.51 (16)	C29—C30—H30	120.5
C41—O5—Sn2	91.36 (14)	C31—C30—H30	120.5
C41—O6—Sn2	91.37 (14)	C32—C31—C30	121.3 (2)
C47—O7—Sn2	132.68 (15)	C32—C31—Br3	120.7 (2)
Sn1—O1w—H1w1	109.5	C30—C31—Br3	118.0 (2)
Sn1—O1w—H1w2	109.5	C31—C32—C33	118.8 (3)
H1w1—O1w—H1w2	109.5	C31—C32—H32	120.6
Sn2—O2w—H2w1	109.5	C33—C32—H32	120.6
Sn2—O2w—H2w2	109.5	C32—C33—C28	121.6 (3)
H2w1—O2w—H2w2	109.5	C32—C33—H33	119.2
C20—N1—C19	117.9 (2)	C28—C33—H33	119.2
C26—N2—C25	118.0 (2)	C35—C34—Sn2	115.37 (17)
C26—N2—Sn2	119.01 (16)	C35—C34—H34A	108.4
C25—N2—Sn2	122.91 (16)	Sn2—C34—H34A	108.4
C45—N3—C46	117.9 (2)	C35—C34—H34B	108.4
C51—N4—C52	117.4 (2)	Sn2—C34—H34B	108.4
C51—N4—Sn1	122.58 (16)	H34A—C34—H34B	107.5
C52—N4—Sn1	119.81 (16)	C40—C35—C36	117.8 (2)
C2—C1—Sn1	114.44 (18)	C40—C35—C34	120.7 (2)
C2—C1—H1A	108.7	C36—C35—C34	121.4 (2)
Sn1—C1—H1A	108.7	C37—C36—C35	121.6 (2)
C2—C1—H1B	108.7	C37—C36—H36	119.2
Sn1—C1—H1B	108.7	C35—C36—H36	119.2
H1A—C1—H1B	107.6	C38—C37—C36	118.5 (2)
C3—C2—C7	117.5 (2)	C38—C37—H37	120.8
C3—C2—C1	121.6 (2)	C36—C37—H37	120.8
C7—C2—C1	120.8 (2)	C37—C38—C39	121.9 (2)
C4—C3—C2	122.0 (2)	C37—C38—Br4	119.7 (2)
C4—C3—H3	119.0	C39—C38—Br4	118.4 (2)
C2—C3—H3	119.0	C38—C39—C40	119.0 (2)
C5—C4—C3	118.8 (2)	C38—C39—H39	120.5
C5—C4—H4	120.6	C40—C39—H39	120.5
C3—C4—H4	120.6	C39—C40—C35	121.2 (2)
C6—C5—C4	121.3 (2)	C39—C40—H40	119.4
C6—C5—Br1	119.0 (2)	C35—C40—H40	119.4
C4—C5—Br1	119.7 (2)	O5—C41—O6	121.3 (2)
C5—C6—C7	118.9 (2)	O5—C41—C42	118.6 (2)
C5—C6—H6	120.6	O6—C41—C42	119.9 (2)
C7—C6—H6	120.6	C43—C42—C46	118.7 (2)
C6—C7—C2	121.5 (2)	C43—C42—C41	120.8 (2)
C6—C7—H7	119.2	C46—C42—C41	120.5 (2)
C2—C7—H7	119.2	C44—C43—C42	119.0 (2)
C9—C8—Sn1	117.10 (17)	C44—C43—H43	120.5
C9—C8—H8A	108.0	C42—C43—H43	120.5
Sn1—C8—H8A	108.0	C43—C44—C45	118.7 (2)
C9—C8—H8B	108.0	C43—C44—H44	120.7
Sn1—C8—H8B	108.0	C45—C44—H44	120.7
H8A—C8—H8B	107.3	N3—C45—C44	123.2 (2)
C14—C9—C10	118.0 (2)	N3—C45—H45	118.4
C14—C9—C8	120.8 (2)	C44—C45—H45	118.4
C10—C9—C8	121.2 (2)	N3—C46—C42	122.5 (2)
C11—C10—C9	121.6 (2)	N3—C46—H46	118.8
C11—C10—H10	119.2	C42—C46—H46	118.8
C9—C10—H10	119.2	O8—C47—O7	126.9 (2)
C12—C11—C10	118.7 (2)	O8—C47—C48	117.0 (2)
C12—C11—H11	120.7	O7—C47—C48	116.1 (2)
C10—C11—H11	120.7	C52—C48—C49	118.4 (2)
C11—C12—C13	121.7 (2)	C52—C48—C47	121.4 (2)
C11—C12—Br2	119.3 (2)	C49—C48—C47	120.1 (2)
C13—C12—Br2	119.0 (2)	C50—C49—C48	118.4 (2)
C12—C13—C14	118.9 (2)	C50—C49—H49	120.8
C12—C13—H13	120.5	C48—C49—H49	120.8
C14—C13—H13	120.5	C51—C50—C49	119.5 (2)
C9—C14—C13	121.2 (2)	C51—C50—H50	120.3
C9—C14—H14	119.4	C49—C50—H50	120.3
C13—C14—H14	119.4	N4—C51—C50	122.6 (2)
O2—C15—O1	121.4 (2)	N4—C51—H51	118.7
O2—C15—C16	120.3 (2)	C50—C51—H51	118.7
O1—C15—C16	118.3 (2)	N4—C52—C48	123.6 (2)
C17—C16—C20	118.2 (2)	N4—C52—H52	118.2
C17—C16—C15	120.8 (2)	C48—C52—H52	118.2
			
C8—Sn1—O1—C15	93.71 (16)	Sn1—O1—C15—O2	−5.3 (3)
C1—Sn1—O1—C15	−89.39 (16)	Sn1—O1—C15—C16	171.3 (2)
O1w—Sn1—O1—C15	4.04 (19)	O2—C15—C16—C17	−175.6 (2)
O3—Sn1—O1—C15	−178.64 (15)	O1—C15—C16—C17	7.8 (4)
O2—Sn1—O1—C15	2.92 (14)	O2—C15—C16—C20	7.7 (4)
N4—Sn1—O1—C15	−177.63 (16)	O1—C15—C16—C20	−169.0 (2)
C8—Sn1—O2—C15	−89.15 (16)	C20—C16—C17—C18	0.1 (4)
C1—Sn1—O2—C15	86.45 (16)	C15—C16—C17—C18	−176.7 (2)
O1w—Sn1—O2—C15	177.98 (16)	C16—C17—C18—C19	0.4 (4)
O3—Sn1—O2—C15	178.66 (15)	C20—N1—C19—C18	0.2 (4)
O1—Sn1—O2—C15	−2.93 (14)	C17—C18—C19—N1	−0.6 (4)
N4—Sn1—O2—C15	−3.62 (18)	C19—N1—C20—C16	0.4 (4)
C8—Sn1—O3—C21	−83.8 (2)	C17—C16—C20—N1	−0.5 (4)
C1—Sn1—O3—C21	100.4 (2)	C15—C16—C20—N1	176.3 (2)
O1w—Sn1—O3—C21	8.0 (2)	Sn1—O3—C21—O4	−26.9 (4)
O1—Sn1—O3—C21	−169.8 (2)	Sn1—O3—C21—C22	151.20 (17)
O2—Sn1—O3—C21	7.4 (3)	O4—C21—C22—C26	−168.6 (2)
N4—Sn1—O3—C21	−170.8 (2)	O3—C21—C22—C26	13.2 (4)
C27—Sn2—O5—C41	87.77 (16)	O4—C21—C22—C23	13.9 (4)
C34—Sn2—O5—C41	−96.97 (16)	O3—C21—C22—C23	−164.4 (2)
O2w—Sn2—O5—C41	−6.97 (19)	C26—C22—C23—C24	−1.4 (4)
O7—Sn2—O5—C41	170.69 (15)	C21—C22—C23—C24	176.2 (2)
O6—Sn2—O5—C41	−4.17 (14)	C22—C23—C24—C25	0.8 (4)
N2—Sn2—O5—C41	175.89 (17)	C26—N2—C25—C24	−1.1 (4)
C27—Sn2—O6—C41	−85.29 (16)	Sn2—N2—C25—C24	174.98 (19)
C34—Sn2—O6—C41	91.24 (16)	C23—C24—C25—N2	0.5 (4)
O2w—Sn2—O6—C41	−178.12 (16)	C25—N2—C26—C22	0.4 (4)
O7—Sn2—O6—C41	−170.32 (15)	Sn2—N2—C26—C22	−175.80 (19)
O5—Sn2—O6—C41	4.15 (14)	C23—C22—C26—N2	0.8 (4)
N2—Sn2—O6—C41	4.23 (18)	C21—C22—C26—N2	−176.8 (2)
C27—Sn2—O7—C47	−97.4 (2)	O2w—Sn2—C27—C28	99.23 (19)
C34—Sn2—O7—C47	87.2 (2)	O7—Sn2—C27—C28	178.8 (2)
O2w—Sn2—O7—C47	−3.4 (2)	O6—Sn2—C27—C28	25.4 (2)
O6—Sn2—O7—C47	−11.0 (3)	O5—Sn2—C27—C28	−29.96 (19)
O5—Sn2—O7—C47	178.46 (19)	N2—Sn2—C27—C28	−103.9 (2)
N2—Sn2—O7—C47	173.3 (2)	Sn2—C27—C28—C33	−84.9 (3)
C27—Sn2—N2—C26	−112.81 (19)	Sn2—C27—C28—C29	94.0 (3)
C34—Sn2—N2—C26	66.78 (19)	C33—C28—C29—C30	0.2 (4)
O2w—Sn2—N2—C26	−18.3 (3)	C27—C28—C29—C30	−178.8 (3)
O7—Sn2—N2—C26	−26.54 (18)	C28—C29—C30—C31	−1.0 (4)
O6—Sn2—N2—C26	155.96 (17)	C29—C30—C31—C32	1.1 (4)
O5—Sn2—N2—C26	156.0 (2)	C29—C30—C31—Br3	−177.9 (2)
C27—Sn2—N2—C25	71.1 (2)	C30—C31—C32—C33	−0.5 (4)
C34—Sn2—N2—C25	−109.3 (2)	Br3—C31—C32—C33	178.5 (2)
O2w—Sn2—N2—C25	165.67 (19)	C31—C32—C33—C28	−0.3 (4)
O7—Sn2—N2—C25	157.4 (2)	C29—C28—C33—C32	0.4 (4)
O6—Sn2—N2—C25	−20.1 (2)	C27—C28—C33—C32	179.4 (3)
O5—Sn2—N2—C25	−20.01 (19)	fO2w—Sn2—C34—C35	69.71 (19)
C8—Sn1—N4—C51	105.0 (2)	O7—Sn2—C34—C35	−10.15 (19)
C1—Sn1—N4—C51	−72.7 (2)	O6—Sn2—C34—C35	143.46 (19)
O1w—Sn1—N4—C51	−164.8 (2)	O5—Sn2—C34—C35	−161.24 (19)
O3—Sn1—N4—C51	−161.9 (2)	N2—Sn2—C34—C35	−87.25 (19)
O1—Sn1—N4—C51	18.55 (19)	Sn2—C34—C35—C40	−75.8 (3)
O2—Sn1—N4—C51	19.1 (2)	Sn2—C34—C35—C36	103.5 (3)
C8—Sn1—N4—C52	−69.34 (19)	C40—C35—C36—C37	0.3 (4)
C1—Sn1—N4—C52	112.9 (2)	C34—C35—C36—C37	−179.0 (2)
O1w—Sn1—N4—C52	20.8 (3)	C35—C36—C37—C38	0.6 (4)
O3—Sn1—N4—C52	23.74 (18)	C36—C37—C38—C39	−0.8 (4)
O1—Sn1—N4—C52	−155.8 (2)	C36—C37—C38—Br4	−178.7 (2)
O2—Sn1—N4—C52	−155.19 (17)	C37—C38—C39—C40	0.0 (4)
O1w—Sn1—C1—C2	−80.2 (2)	Br4—C38—C39—C40	177.9 (2)
O3—Sn1—C1—C2	−159.5 (2)	C38—C39—C40—C35	1.0 (4)
O1—Sn1—C1—C2	48.3 (2)	C36—C35—C40—C39	−1.2 (4)
O2—Sn1—C1—C2	−6.8 (2)	C34—C35—C40—C39	178.2 (2)
N4—Sn1—C1—C2	122.5 (2)	Sn2—O5—C41—O6	7.5 (3)
Sn1—C1—C2—C3	83.1 (3)	Sn2—O5—C41—C42	−167.4 (2)
Sn1—C1—C2—C7	−94.6 (3)	Sn2—O6—C41—O5	−7.5 (3)
C7—C2—C3—C4	0.8 (4)	Sn2—O6—C41—C42	167.3 (2)
C1—C2—C3—C4	−177.0 (3)	O5—C41—C42—C43	7.2 (4)
C2—C3—C4—C5	0.0 (4)	O6—C41—C42—C43	−167.8 (2)
C3—C4—C5—C6	−1.0 (4)	O5—C41—C42—C46	−174.7 (2)
C3—C4—C5—Br1	178.1 (2)	O6—C41—C42—C46	10.3 (4)
C4—C5—C6—C7	1.2 (4)	C46—C42—C43—C44	0.8 (4)
Br1—C5—C6—C7	−177.9 (2)	C41—C42—C43—C44	178.8 (2)
C5—C6—C7—C2	−0.4 (4)	C42—C43—C44—C45	0.7 (4)
C3—C2—C7—C6	−0.6 (4)	C46—N3—C45—C44	0.2 (4)
C1—C2—C7—C6	177.2 (3)	C43—C44—C45—N3	−1.2 (4)
O1w—Sn1—C8—C9	−76.71 (19)	C45—N3—C46—C42	1.4 (4)
O3—Sn1—C8—C9	2.77 (19)	C43—C42—C46—N3	−1.8 (4)
O1—Sn1—C8—C9	154.96 (19)	C41—C42—C46—N3	−179.9 (2)
O2—Sn1—C8—C9	−149.98 (19)	Sn2—O7—C47—O8	18.4 (4)
N4—Sn1—C8—C9	80.54 (19)	Sn2—O7—C47—C48	−160.24 (16)
Sn1—C8—C9—C14	81.5 (3)	O8—C47—C48—C52	177.4 (2)
Sn1—C8—C9—C10	−99.6 (3)	O7—C47—C48—C52	−3.8 (4)
C14—C9—C10—C11	0.6 (4)	O8—C47—C48—C49	−4.3 (4)
C8—C9—C10—C11	−178.3 (2)	O7—C47—C48—C49	174.5 (2)
C9—C10—C11—C12	0.0 (4)	C52—C48—C49—C50	1.5 (4)
C10—C11—C12—C13	−0.3 (4)	C47—C48—C49—C50	−176.9 (2)
C10—C11—C12—Br2	178.4 (2)	C48—C49—C50—C51	−0.3 (4)
C11—C12—C13—C14	0.1 (4)	C52—N4—C51—C50	2.1 (4)
Br2—C12—C13—C14	−178.66 (19)	Sn1—N4—C51—C50	−172.4 (2)
C10—C9—C14—C13	−0.9 (4)	C49—C50—C51—N4	−1.6 (4)
C8—C9—C14—C13	178.1 (2)	C51—N4—C52—C48	−0.8 (4)
C12—C13—C14—C9	0.5 (4)	Sn1—N4—C52—C48	173.81 (19)
Sn1—O2—C15—O1	5.3 (3)	C49—C48—C52—N4	−1.0 (4)
Sn1—O2—C15—C16	−171.3 (2)	C47—C48—C52—N4	177.4 (2)

### Hydrogen-bond geometry (Å, °)

**Table:**

*D*—H···*A*	*D*—H	H···*A*	*D*···*A*	*D*—H···*A*
O1w—H1w1···O4	0.84	1.94	2.557 (2)	129
O1w—H1w2···N3^i^	0.84	2.06	2.663 (3)	128
O2w—H2w2···O8	0.84	1.96	2.563 (2)	128
O2w—H2w1···N1^ii^	0.84	2.26	2.690 (3)	112

Symmetry codes: (i) *x*, *y*−1, *z*; (ii) *x*, *y*+1, *z*.
